# One-carbon metabolism: An aging-cancer crossroad for the gerosuppressant metformin

**DOI:** 10.18632/aging.100523

**Published:** 2013-01-26

**Authors:** Javier A. Menendez, Jorge Joven

**Affiliations:** ^1^ Metabolism & Cancer Group, Translational Research Laboratory, Catalan Institute of Oncology-Girona (ICO-Girona), Girona, Spain; ^2^ Molecular Oncology, Girona Biomedical Research Institute (IDIBGi), Girona, Spain; ^3^ Unitat de Recerca Biomèdica (URB-CRB), Institut d'Investigació Sanitaria Pere i Virgili (IISPV), Universitat Rovira i Virgili, Reus, Spain

**Keywords:** metformin, one-carbon metabolism, nucleotides, purines, AMP, ATP, AMPK, mTOR, gerosuppression, aging, cancer

## Abstract

The gerosuppressant metformin operates as an efficient inhibitor of the mTOR/S6K1 gerogenic pathway due to its ability to ultimately activate the energy-sensor AMPK. If an aging-related decline in the AMPK sensitivity to cellular stress is a crucial event for mTOR-driven aging and aging-related diseases, including cancer, unraveling new proximal causes through which AMPK activation endows its gerosuppressive effects may offer not only a better understanding of metformin function but also the likely possibility of repositioning our existing gerosuppressant drugs. Here we provide our perspective on recent findings suggesting that *de novo* biosynthesis of purine nucleotides, which is based on the metabolism of one-carbon compounds, is a new target for metformin's actions at the crossroads of aging and cancer.

It is perhaps not surprising that the cellular energy sensor adenosine monophosphate (AMP)-activated protein kinase (AMPK), a critical suppressor of the mTOR gerogene [1-17], has been once again highlighted as a conserved life span modulator linking bioenergetics, metabolism, and longevity [12-22]. What is certainly surprising is the *proximate* causation through which AMPK activation has now been shown to enable its pro-longevity effects. When searching for mutations capable of disrupting energy balance in metabolically active tissues and slowing aging in the fruit fly *Drosophila melanogaster*, Stenesen and colleagues [23] recently found that the inactivation of genes coding for enzymes involved in the *de novo* synthesis of the purine nucleotide AMP demonstrated the strongest pro-longevity effects. Interestingly, mutations in AMP biosynthetic enzymes capable of significantly extending the *Drosophila* lifespan impacted cellular bioenergetics by unexpectedly increasing the AMP:ATP and ADP:ATP ratios, thus counter intuitively mimicking the effects of energy depletion (e.g., dietary restriction), despite disrupting AMP biosynthesis [23,24]. AMPK, the cellular fuel gauge whose activity becomes significantly increased in long-lived flies, detects such energy imbalances to causally channel longevity effects resulting from genetically impaired *de novo* AMP synthesis. While the expression of a dominant-negative form of AMPK prevented the lifespan increases driven by heterozygous mutations in AMP biosynthetic enzymes, animals engineered to specifically exhibit AMPK gain-of-function in metabolic tissues also had lifespan increases equivalent to those observed in long-lived fly mutants. Therefore, enhanced AMPK activity appears to be sufficient to fully recapitulate the ability of AMP biosynthesis pathway mutations to increase the AMP:ATP ratio and longevity.

In the novel scenario illustrated by Stenesen and colleagues [23], it reasonably follows that small molecule drugs capable of mimicking the energy imbalance imposed by mutations in the AMP biosynthesis pathway may be expected to increase healthy life spans by activating AMPK. Moreover, given that AMPK is a crucial gerosuppressor (and tumor-suppressor) that impedes mTOR-driven geroconversion (and mTOR-driven malignant transformation) [1-17], small molecules capable of activating AMPK by altering the *de novo* synthesis of purine nucleotides such as AMP should be expected to not only inhibit the pro-aging activity of mTOR gerogenes but also prevent aging-related diseases, such as cancer. The antidiabetic biguanide metformin may fulfill all of these requirements. First, epidemiological, preclinical, and clinical evidence from the last five years has demonstrated the multi-faceted capabilities of metformin in preventing and treating human carcinomas [25-35]. Second, metformin, independently of the insulin-signaling pathway, has been noted to significantly extend the healthy lifespan of not only non-diabetic mice but also the nematode *Caenorhabditis elegans* [36-42]. AMPK, which is activated in mammals by metformin treatment, has also been found to be an essential molecular operative for metformin healthspan benefits in *C. elegans* [42], thus suggesting that the metformin gerosuppressant activity largely depends on its ability to engage the same metabolic sensor, i.e., AMPK, which is highly conserved across phyla. Third, metformin prevents cancer and extends the lifespan of cancer-prone rodent strains. Moreover, metformin can also prolong lifespan without affecting cancers in non-cancer-prone rodent strains [36-41]. Although the latter discrepancy may suggest that metformin could delay aging (and prolong life) by mechanisms unrelated to its ability to suppress cancer, it may not if this discrepancy simply relies on a cancer-related enhancement of common proximate anti-aging mechanisms by which metformin can activate the gerosuppressor/tumor suppressor AMPK. One such mechanism may be one-carbon metabolism that drives the *de novo* synthesis of purine nucleotides (e.g., AMP).

It is well known that the relative contribution of nucleotide biosynthesis to nucleotide pool maintenance via the *de novo* and salvage pathways significantly varies in different cells and tissues. Proliferating cells, including cancer cells, usually require a functional *de novo* pathway to sustain their increased nucleotide demands. Indeed, this activity is the basis for the use of antifolate drugs in chemotherapy against cancer cells, which generally have higher DNA turnover. Crucially, a recently identified metabolomic fingerprint of human cancer cells treated with metformin revealed for the first time its previously unrecognized ability to significantly impair one-carbon metabolism and the *de novo* biosynthesis of purine nucleotides in a manner that is functionally similar but mechanistically different than that of the antifolate class of chemotherapy drugs [43]. Of note, the ability of metformin to activate the AMPK metabolic tumor suppressor and inhibit cancer cell growth was notably prevented when the salvage branch of purine biosynthesis was promoted by exogenous supplementation with the pre-formed substrate hypoxanthine, a spontaneous deamination product of the purine adenine. Remarkably, Stenesen and colleagues [23] similarly found that dietary supplementation with adenine, the pre-formed substrate of AMP biosynthesis, not only markedly reversed the lifespan extension of AMP biosynthesis mutants but also the pro-longevity effects of dietary restriction. The recognition of *de novo* AMP biosynthesis, adenosine nucleotide ratios, and AMPK as determinants of the *Drosophila* adult lifespan and the finding that the anti-cancer activity of metformin could be explained in terms of the secondary activation of AMPK following the alteration of the essential carbon flow that leads to the *de novo* synthesis of purines both strongly suggest that the flow of one-carbon groups governing the *de novo* biosynthesis of purines could represent a crucial metformin-targeted intersection of aging with cancer (Fig. 1).

**Figure 1:**
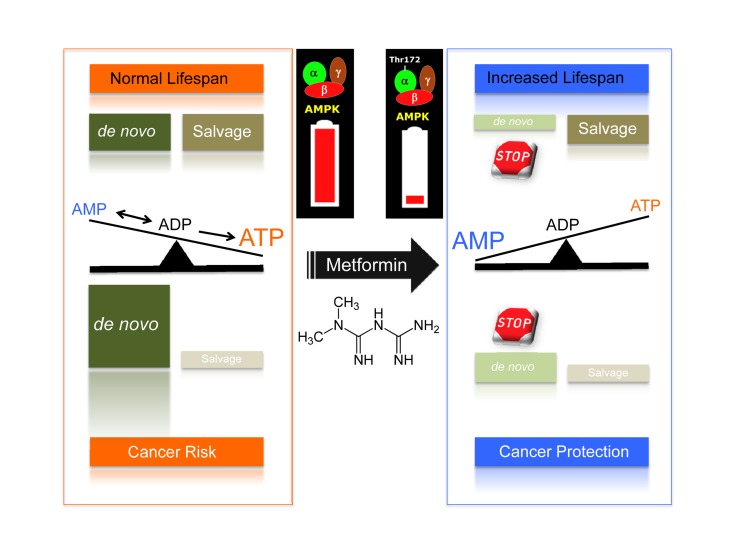
*De novo* biosynthesis of purine nucleotide at the crossroads of aging and cancer: A new target for the gerosuppressant metformin

Because a ubiquitous event in cancer metabolism is the early, constitutive activation of one-carbon metabolism and because *de novo* nucleotide biosynthesis may influence cancer mortality due to its critical role in DNA synthesis and methylation, the repeatedly suggested reduction in cancer risk and mortality of diabetic patients chronically treated with metformin may therefore represent an unintended metronomic chemotherapy approach targeting the differential utilization of *de novo* one-carbon metabolism by malignant and non-malignant cells [43]. In light of the findings by Stenesen and colleagues [23], it may be reasonable to suggest that metformin treatment may silently operate not only to eliminate genetically damaged, initiated, or malignant cells addicted to higher nucleotide concentrations but also activate the gerosuppressant activity of AMPK by unbalancing the *de novo* biogenesis of the purine AMP in metabolically active tissues (Fig. 1). It may be argued that the ability of metformin to activate AMPK following the inhibition of one-carbon metabolism indicates its teratogenic potential [43,44]. Although one study reported no alterations in embryonic growth and no major malformations during mouse embryogenesis, it is noteworthy that the metformin analog phenformin, an AMPK activator that is more potent than metformin, remarkably produced embryolethality and embryo malformations, including neural tube closure defectsand craniofacial hypoplasia [44]. Future studies may elucidate whether phenformin has a stronger inhibitory effect on *de novo* purine biosynthesis compared with metformin.

Nevertheless, we should acknowledge that while high doses of metformin have been reported to increase the lifespan of *C. elegans* in an AMPK-dependent manner [42], this metformin effect could not be observed in fruit flies [45]. Thus, while AMPK activation increases lifespan in *Drosophila*, metformin supplementation does not. Forthcoming studies should determine whether the lack of equivalence between feeding metformin and activating AMPK may be due to either off-target detrimental metformin effects or the detrimental effects of systemically activating AMPK in relevant *versus* non-relevant tissues for lifespan extension [24]. In this regard, it should also be considered that while previous studies in fibroblasts and rat hepatoma cells have shown that AMPK activation by metformin occurred by mechanisms other than changes in the cellular AMP:ATP ratio [46], recent evidence in primary hepatocytes has revealed that metformin activates AMPK by decreasing the cellular energy status *via* a significant rise in the cellular AMP:ATP ratio [47]. Moreover, metformin has been reported to mimic a low-energy AMPK-activating state by increasing AMP levels through the inhibition of AMP deaminase (AMPD) in skeletal muscle cells and the development of fatty liver [48,49]. Curiously, when Stenesen and colleagues [23] tested the longevity effects of an insertional mutation in AMPD that catalyzes the hydrolytic deamination of AMP into inosine monophosphate, i.e., the opposite direction of the longevity genes adenylsuccinate synthetase, adenylsuccinate lyase, adenosine kinase, and adenine phosphoribosyltransferase, they failed to observe any effects on lifespan. Whether the metformin ability to directly [48] or indirectly inhibit AMPD, such as through the accumulation of intermediates during the folate-dependent metabolism of one carbon unit [43], could counteract the longevity induced by AMPK activation certainly merits further exploration.

The molecular mechanism(s) through which the gerosuppressant metformin could increase life span and delay tumor formation and progression remain unclear. Most studies have focused on *ultimate causes*, which mostly involve the reasons why metformin has beneficial effects. An ever-growing experimental body of evidence strongly suggests that metformin operates as an efficient inhibitor of the mTOR/S6K1 gerogenic pathway due to its ability to ultimately activate the AMPK energy-sensor in a cell-autonomous manner. If an aging-related decline in the AMPK sensitivity to cellular stress is a crucial event for mTOR-driven aging and aging-related diseases, including cancer, it is now time to explore molecular events that primarily involve the "how" questions; unraveling new *proximal causes* through which AMPK activation endows its gerosuppressive effects may offer not only a better understanding of metformin function but also the likely possibility of repositioning our existing gerosuppressant drugs.
